# Tropical soils degraded by slash‐and‐burn cultivation can be recultivated when amended with ashes and compost

**DOI:** 10.1002/ece3.3104

**Published:** 2017-06-12

**Authors:** Justine Marie Gay‐des‐Combes, Clara Sanz Carrillo, Bjorn Jozef Maria Robroek, Vincent Eric Jules Jassey, Robert Thomas Edmund Mills, Muhammad Saleem Arif, Leia Falquet, Emmanuel Frossard, Alexandre Buttler

**Affiliations:** ^1^ ECOS Laboratory École Polytechnique Fédérale de Lausanne Lausanne Switzerland; ^2^ Swiss Federal Institute for Forest Snow and Landscape Research Lausanne Switzerland; ^3^ Escuela Técnica Superior de Ingeniería Agronómica Alimentaria y de Biosistemas Universidad Politécnica de Madrid Madrid Spain; ^4^ Biological Sciences University of Southampton Southampton UK; ^5^ Lancaster Environment Centre Lancaster University Lancaster UK; ^6^ Department of Environmental Sciences and Engineering Government College University Faisalabad Faisalabad Pakistan; ^7^ Plant Nutrition Group Institute for Agricultural Sciences ETH Zurich Lindau Switzerland; ^8^ Laboratoire de Chrono‐Environnement Université de Franche‐Comté Besançon France

**Keywords:** crop yield, deforestation, microbial activity, organic matter, soil fertility, structural equation model

## Abstract

In many tropical regions, slash‐and‐burn agriculture is considered as a driver of deforestation; the forest is converted into agricultural land by cutting and burning the trees. However, the fields are abandoned after few years because of yield decrease and weed invasion. Consequently, new surfaces are regularly cleared from the primary forest. We propose a reclamation strategy for abandoned fields allowing and sustaining re‐cultivation. In the dry region of south‐western Madagascar, we tested, according to a split‐plot design, an alternative selective slash‐and‐burn cultivation technique coupled with compost amendment on 30–year‐old abandoned fields. Corn plants (*Zea mays* L.) were grown on four different types of soil amendments: no amendment (control), compost, ashes (as in traditional slash‐and‐burn cultivation), and compost + ashes additions. Furthermore, two tree cover treatments were applied: 0% tree cover (as in traditional slash‐and‐burn cultivation) and 50% tree cover (selective slash‐and‐burn). Both corn growth and soil fertility parameters were monitored during the growing season 2015 up to final harvest. The amendment compost + ashes strongly increased corn yield, which was multiplied by 4–5 in comparison with ashes or compost alone, reaching 1.5 t/ha compared to 0.25 and 0.35 t/ha for ashes and compost, respectively. On control plots, yield was negligible as expected on these degraded soils. Structural equation modeling evidenced that compost and ashes were complementary fertilizing pathways promoting soil fertility through positive effects on soil moisture, pH, organic matter, and microbial activity. Concerning the tree cover treatment, yield was reduced on shaded plots (50% tree cover) compared to sunny plots (0% tree cover) for all soil amendments, except ashes. To conclude, our results provide empirical evidence on the potential of recultivating tropical degraded soils with compost and ashes. This would help mitigating deforestation of the primary forest by increasing lifespan of agricultural lands.

## 1 INTRODUCTION

Tropical forests support a large and unique biodiversity, but are threatened worldwide by rapid deforestation due to their conversion into agricultural lands. Deforestation rates are particularly alarming in Madagascar, a country which is pointed out as one of the eight leading global biodiversity hotspots in terms of endemic species (Myers, Mittermeier, Mittermeier, da Fonseca, & Kent, 2000). On the south‐western coast of the island, in Menabe region, deforestation annual rate of the dry deciduous forest has been estimated at 2.6% between 2008 and 2010, *that is,* a loss of 1,820 ha per year (Zinner et al., 2014). At that rate, the forest and most, if not all, its endemic species, will have entirely disappeared by 2050 (Zinner et al., 2014).

In Madagascar, slash‐and‐burn agriculture is the main driver of deforestation (Dirac Ramohavelo, 2009; Genini, 1996). In this traditional, widespread technique, forest is converted to agricultural land by cutting and subsequently burning trees, the ashes being used to amend the soil. After few years (between 2–4 years, depending on the regions) of repetitive cultivation, crop yield decreases while weeds invade the agricultural fields. Farmers then generally abandon their fields and convert new surfaces of primary forest into agricultural fields. As a consequence, the primary forest area decreases progressively, while degraded land increases. In Madagascar, extreme measures like anti‐fire policies were applied for over one‐hundred years without much success in putting halt to deforestation (Jarosz, 1993; Kull, 2002). Farmers still practice shifting cultivation, even against the law, because it is most convenient to them (Kull, 2002), but also because this method is embedded within a strong spiritual and social background (Desbureaux & Brimont, 2015; Hume, 2006; Jarosz, 1993; Scales, 2012).

Slash‐and‐burn agriculture modifies the physicochemical properties of the soil (Are, Oluwatosin, Adeyolanu, & Oke, 2009; Béliveau et al., 2015; Demeyer, Voundi Nkana, & Verloo, 2001; Thomaz, Antoneli, & Doerr, 2014). Ashes are strongly alkaline, which reduces soil acidity, boosts microbial activity and increases soil nutrient availability (Ohno & Erich, 1990). This is particularly useful in tropical acid soils, as it favors plant growth. The effects on the soil nutrients are, however, short term for some highly soluble elements subject to leaching, for example, potassium (K), calcium (Ca), or magnesium (Mg) (Demeyer et al., 2001; Ulery, Graham, & Amrhein, 1993). Additionally, ashes push the stoichiometry of soil nutrients toward nitrogen (N) limitation, as after combustion, the nitrogen contained in tree leaves is volatilized (Ohno & Erich, 1990). Hence, ashes fertilization is not appropriate for long‐term cultivation, occasioning soil nutrient exhaustion after repetitive cultivation during few years (Folberth, Yang, Gaiser, Abbaspour, & Schulin, 2013). Also, by cutting and burning the trees, field surface remains bare, with no protection against strong winds and rains. Tropical heavy rains can lead to soil saturation and the formation of free water on the surface, which further causes soil erosion even on slightly sloping terrains. Such soil erosions are problematic for maize cultivations as a single cyclonic storm can reduce corn yields by more than 75% (Gay‐des‐Combes et al., 2017).

Regeneration of the forest on most tropical degraded soils requires a minimal of 10 years of fallow period (Thomaz et al., 2014). However, in western Madagascar, it happens that the soil never recovers from such degradation, leading to large abandoned areas where the ecosystem shifts from forests to savannahs (Raharimalala et al., 2010). Clearly, re‐cultivating the abandoned surfaces is urgent and would safeguard agricultural land, while simultaneously reducing the pressure on the primary forest. However, to be successful, the re‐cultivation needs alternative techniques that both slow down, or even stop, soil degradation and leave intact the socially and cultural embedded practice of slash‐and‐burn techniques. Indeed, maintaining the use of fire when developing alternative techniques might be non‐negotiable to convince farmers to change their agricultural practices. In this context, the use of biochar, as an alternative to the ashes, could be a promising solution in terms of soil amendment with burnt wood. Biochar contains “black carbon” which are residues of incomplete combustion of organic material. Its structure, composed of aromatic cycles, is chemically and microbiologically stable, leading, when amended to soils, to the formation of persistent organic matter over years (Glaser, Haumaier, Guggenberger, & Zech, 2001). Thus, biochar amendment boosts the overall quality of soil by improving its nutrient status, its water holding capacity, and its microbial activity (Hass et al., 2012; Laird et al., 2010; Mitchell, Simpson, Soong, & Simpson, 2015). However, biochar is hardly reproducible on a frequent basis as its efficiency strongly depends from its final polyaromatic structure determined by the type of material used and its combustion temperature (Butnan, Deenik, Toomsan, Antal, & Vityakon, 2015; Reed, Chadwick, Hill, & Jones, 2017). Furthermore, farmers in developing countries are not always willing to accept, let alone being able to pay, the costs for the application of biochar (Cernansky, 2015).

Although ashes and biochar are closely related material and could have similar effects on the soil fertility (Reed et al., 2017), the main problem of traditional wood ashes compared to biochar is the absence of solid structure stabilizing nutrients in the soil. Here, we suggest that an addition of organic matter, such as compost, to wood ashes could play this role (Bougnom et al., 2010). Compost enhances both water‐ and nutrient‐holding capacity of the soil (Zhang et al., 2016). Therefore, combining compost and ashes may play a significant role for tropical soil security by mitigating nutrients leaching (Agegnehu, Nelson, & Bird, 2016). Besides, compost is also known to increase plant yield through the addition of nutrients (Abdel‐Sabour & El‐Seoud, 1996; Mbau, Karanja, & Ayuke, 2014; Zhang et al., 2016). Additionally, letting some trees within the fields could also rise the soil organic matter content through littering (Nair, 2013). Furthermore, trees prevent soil erosion and contribute to maintaining soil microbial community (Carrière, Letourmy, & McKey, 2002). Thus, we tested a selective slash‐and‐burn agriculture (where some trees are intentionally not cut), coupled with compost amendment. We believe that this system could lead to significant improvements of soil fertility and crop yields, while respecting traditional slash‐and‐burn method. We specifically aimed at answering two questions addressing the effects of the aforementioned technique. First, do a partial remaining tree cover and compost amendment increase corn yield, as compared to traditional slash‐and‐burn agriculture? Second, what are the respective contributions of tree cover, ashes and compost to soil organic matter, its nutrient status and microbial activity? We hypothesized that compost amendment and tree cover will increase organic matter, microbial biomass, as well as N and P contents of the soil. Ash, on the other hand, should rise soil pH, which in turn promotes the mineralization of organic matter. Because of soil fertility improvement, we also predicted that corn amended with both ashes and compost will show the highest yield.

## 2 MATERIALS AND METHODS

### 2.1 Study site

Field sampling was conducted in the dry deciduous forest of Kirindy (central Menabe) near the village of Beroboka (20°00′22.3″S, 44°35′06.1″E). The nearest large city is Morondava, located 30 km south. Beroboka was selected because it is strongly affected by deforestation due to slash‐and‐burn cultivation (Dirac Ramohavelo, 2009) and it offers a large area (about 1,000 ha) of abandoned fields, thus allowing experimentations on degraded soils. The climate is tropical, but with a long dry season from April to September. Mean annual rainfall is about 800 mm, but varies from 250 to 1,400 mm depending on the year and cyclonic events (Sorg & Rohner, 1996). Temperatures are high year round and average at 24.7°C (Sorg & Rohner, 1996).

### 2.2 Plot selection and experimental design

Three sites, 300 m distant from each other, were selected on 30‐year‐old abandoned fields with comparable vegetation structure, mainly small shrubs and a few tall trees. The soils at the three sites were ferruginous soils, corresponding to the Lixisols after the World Reference Base for Soil Resources (Raharimalala et al., 2010). The soils had similar nutrient concentrations (Table 1), were yellowish red (5YR 5/8, Munsell color code), and composed of about 15% clay, 10% silt, and 75% of sand. On half of the surface in each site all vegetation was removed (0% tree cover; treatment “sunny’), and on the other half, shrubs and some trees were removed (50% tree cover, treatment “shaded”). Within each tree cover treatment, four 3 × 3 m plots were allocated randomly to one of the following treatments: control (Ctr), ashes (Ash), compost (Comp), and ashes mixed with compost (CoAs). This led to a split‐plot design with eight plots in each experimental site (four per tree cover treatment), 24 in total. The treatments containing ashes (Ash, CoAs) were prepared with the help of local farmers, in order to follow closely the traditional slash‐and‐burn practices, in particular the quantities per surface unit. They resulted from gathering and burning the wood from the cut trees at each desired plot location, and subsequent application of a 1.5‐cm ash layer on the soil.

**Table 1:** Mean values with standard deviations for some chemical parameters of the soil found in the experimental fields and for the ashes and compost used in the experiment

Material	Density (kg dry wt/L)	pH	CEC (cmol/kg)	O.M. (g/kg)	Ctot (g/kg)	Ntot (g/kg)	Ninorg (mg/kg)	Ptot (mg/kg)	P resin (mg/kg)	K ex (mg/kg)
Control soil	1.4 ± 0.1	5.8 ± 0.2	5.1 ± 0.8	57.2 ± 4.2	10.7 ± 2.9	0.95 ± 0.03	2.1 ± 1.0	71.9 ± 5.1	2.3 ± 0.5	64.2 ± 3.3
Compost	0.6 ± 0.1	7.5 ± 0.1	36.8 ± 0.5	685.8 ± 35.4	253.9 ± 20.5	18.2 ± 1.2	189.0 ± 12.3	1,553.9 ± 106.1	162.9 ± 12.0	4,331.2 ± 153.8
Ashes	1.1 ± 0.2	8.6 ± 0.2	15.2 ± 1.1	371.7 ± 25.2	123.6 ± 5.7	7.4 ± 0.4	29.0 ± 3.1	640.0 ± 23.2	11.9 ± 1.3	2,630.0 ± 65.1

At the start of the rainy season, on 27 December 2014, three corn seeds were buried 10 cm deep at 10 different spots within each plot. We used the main local corn variety called “vonivony,” with a growth cycle of 110 days. At a later stage, a thinning took place and only one plant was left in each plantation hole, leaving 10 plants per plot, that is, 1.1 plant m^−2^, comparable to usual field density in the region. In the compost treatments (Comp and CoAs), 1 L of compost was added in each plantation hole when burying the seeds. The compost was produced and matured during the 8 months prior to the experiment with an equal volume of branches and leaves coming from three local trees (*Poupartia sylvatica*,* Tarenna sericea,* and *Fernandoa madagascariensis*). Only small branches were collected in order to leave the trees undamaged. Besides, these tree species were chosen because they have a high content of nutrients and are very abundant in secondary forest successions, preventing further degradations of the primary forest (Raharimalala, 2011; Raharimalala, Buttler, Schlaepfer, & Gobat, 2012).

### 2.3 Soil and corn sampling

Soil moisture was measured at 10 cm in each plot halfway the growing season (i.e., 15 March 2015), using a TDR probe (FieldScout 100). On 29 March, at the end of the growing season and before plant senescence started, plant final heights were measured. On 27 April, once plants started to dry, shoot and bulk grains were collected. Shoot and grains were sun dried during 3 days, until constant weight, and measured. As the plant density in the experimental plots was similar to the density found in surrounding corn fields (Gay‐des‐Combes et al., 2017), dry weight of bulk grains per plot was converted to grain yield per ha. Finally, in the same time than corn harvest, on 27 April, three soil samples (c. 100 g) were collected, within each plot (0–10 cm depth), pooled together to reduce heterogeneity, sun dried during 3 days, until constant weight, and finally sieved at 2 mm.

### 2.4 Laboratory analyses

#### 2.4.1 Plant nutrient content and uptake

Nitrogen (N), phosphorus (P), and potassium (K) content of the corn leaves and grains were assessed by digesting the plant samples as described by (Wolf, 1982). Potassium (K) was determined by atomic absorption spectrophotometry (Solaar 969, ThermoOptek). Total nitrogen (N) and phosphorus (P) concentrations were determined by, respectively, the blue‐indophenol method and the molybdovanadate method using a continuous flow autoanalyser (FlowSys, Systea). Plant uptake in aerial parts was calculated as the sum of the product of leaf and grain nutrient content and respective leaf and grain dry weight.

#### 2.4.2 Soil, ashes, and compost parameters

All measures were performed on the initial soil of the experimental sites before the experiment started, the ashes, and compost (at maturation) used in the experiment, and soil samples collected after the experiment. Total C and N were measured on milled soil with a standard CHN analyser (Dumas method). The pH was assessed in a 1:2.5 soil:water suspension (v:v) (Allen, 1989). Cation exchange capacity (CEC), exchangeable K, and inorganic nitrogen (NO_3_ and NH_4_) were measured by extraction: CEC and K with Cobalt‐hexamine solution (Co(NH_3_)Cl_3_ 0.0166 mol/L) and inorganic nitrogen with KCl (1 mol/L). Later, inorganic nitrogen forms were analyzed colorimetrically using a continuous flow analyzer. CEC and exchangeable K concentrations were read on a plasma atomic emission spectrometer (Shimadzu ICPE‐9000). Soil phosphorus availability was assessed on anion‐exchange resins (Hedley, Steward, & Chauhan, 1982), and the concentrations were measured colorimetrically using the malachite green method and placing the samples in a Shimadzu 1800 UV–vis spectrophotometer (Ohno & Zibilske, 1991). Finally, organic matter was measured through loss of ignition at 450°C.

#### 2.4.3 Microbial biomass and soil enzyme activity

Microbial C biomass was measured with a chloroform fumigation followed by 0.5 mol/L K_2_SO_4_ extraction (Vance, Brookes, & Jenkinson, 1987)**.** As a proxy for soil activity, we measured the activity of four enzymes involved in C, N, and P cycling (Sinsabaugh et al., 2008). We quantified the relative activity (i.e., enzyme activity under optimal and saturating substrate conditions) of extracellular enzymes responsible for the hydrolysis of one peptide (Leucine amino‐peptidase, LAP, N cycle), two carbohydrates (β‐glucosidase, BG; Chitinase, CHI, C, and N cycle, respectively), and one phosphatase (alkaline phosphatase, AP, P cycle; all substrates supplied Sigma‐Aldrich Switzerland). Enzymes were determined on 1 g (wet weight) aliquots of soil and analyzed in microplates following Jassey et al. (2016).

### 2.5 Statistical analyses

The effects of ashes, compost, and tree cover on soil parameters and plant morphological traits were analyzed using split‐plot analysis of variance (ANOVA). The effect of the different treatments was tested while the site effect was corrected by adding “site” as a random variable (block). Tukey's post hoc analyses were used to determine the differences among amendment treatments. When required, values were log‐transformed before the analyses to satisfy the assumption of normality and homogeneity of variance**.** These analyses were performed in *R* (R Development Core Team, 2013)**.**

The analyses described above assess well the responses of soil descriptors and plant traits to amendment treatments. However, the underlying drivers of corn yield need to be determined. To investigate whether the tree cover, compost, and ashes directly drive the soil fertility and consequently corn yield, we established a structural equation model (Grace, Anderson, Olff, & Scheiner, 2010; Grace et al., 2012). Following current concepts of microbial processes and soil fertility, we built an a priori conceptual model of hypothesized causal relationships within a path diagram (Table S2; Grace, Adler, Stanley Harpole, Borer, & Seabloom, 2014). Microbial stock and activity were represented by carbon microbial biomass as it was strongly correlated to acid phosphatase (*r* = .63, *p *=* *.0012), beta‐glucosidase (*r* = .88, *p *<* *.001), as well as chitinase activity (*r* = .86, *p *<* *.001). To increase model stability, the variables “ash” and “tree cover” were removed from the model, as ash and pH were strongly correlated (*r* = .77, *p *<* *.001), and tree cover and humidity too (*r* = .55, *p *=* *.005). The adequacy of the model was assessed using chi‐square tests, root mean square error of approximation index (RMSEA), Akaike value (AIC), standardized root mean square residual index (SRMR), and good fitness index (GFI). Adequate model fits showed nonsignificant differences when comparing the predicted and observed correlation matrices (chi‐square tests with *p *>* *.05) and are indicated by RMSEA < 0.05, by lower AIC, SRMR < 0.05 and GFI > 0.95 (Grace et al., 2010). The structural equation model was achieved with the *R* package *sem* (Fox, 2006).

## 3 RESULTS

The soils of the experimental sites were moderately acid (pH = 5.8) and had a limited content of organic matter and a low CEC (O.M. = 57.2 g/kg, CEC = 5.1 cmol/kg, Table 1). The ashes and the compost used as soil amendments in the experiment contained about 5 and 20 times more nutrients than the control soil, respectively (Table 1). Ashes were basic (pH = 8.6), while compost was close to neutral (pH = 7.5, Table 1). Although ashes are generally mostly mineral, they still contained 37% of organic matter (Table 1).

### 3.1 Corn growth, yield, and nutrient allocation

**Figure 1:**
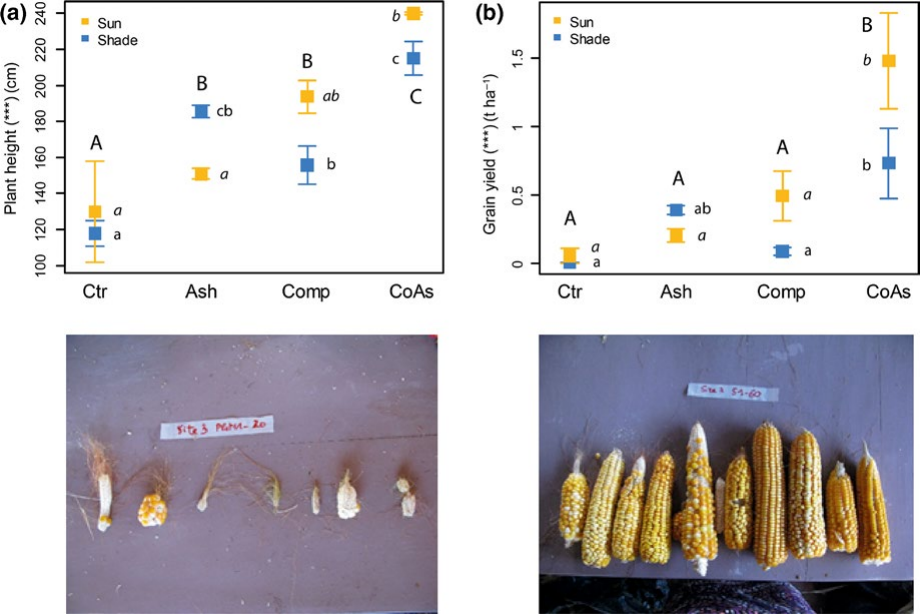
Mean (±standard errors) plant height (a) and grain yield (b) at harvest for each soil amendment treatment on both sunny and shaded plots of the experiment. Asterisks indicate significance in the variable variation according to the treatments (***, *p* < .001; **, *p* < .01; *, *p* < .05; ^.^, *p* < .1). Different letters denote significant differences between soil amendment treatments (Tukey's tests, *p *≤* *.05). Capital letters stand for the overall treatment; normal lower case letters for the shaded plots and italic lower case letters for the sunny plots. (c) Harvested corn cobs on a sunny “Ctr” plot. (d) Harvested corn cobs on a sunny “CoAs” plot

While plant height and yield were similar between compost and ash amendments (Ash, Comp) for both sunny and shaded conditions, yield was multiplied in the compost and ash mixture (CoAs) under sunny conditions, that is, the dry bulk grain weight, by six (from 0.25 to 1.5 t/ha) and increased plant height by 60% (from 150 to 240 cm) compared to traditional slash‐and‐burn method (Ash) (Figure 1). “CoAs” treatment also led to a threefold increase in grain yield, as compared to “Comp” treatment (from 0.5 to 1.5 t/ha), while plant height increased by 26% (from 190 to 240 cm). Lower plant heights and corn yields were found on the shaded part of the experiment, except with “Ash” treatment. In the latter case, plant height increased by 20% (from 155 to 185 cm) and corn yield almost doubled (from 0.25 to 0.4 t/ha; Figure 1).

Tree cover did not affect plant height, nor yield by itself. Yet, the interaction between tree cover and soil amendment was significant (plant height, *p *=* *.0367; corn yield, *p *=* *.051, Table S1). On control plots (Ctr), corn yield was close to zero for both sunny and shaded conditions (Figure 1). We observed similar trends for the nutrient uptakes. The plant uptake was the largest on sunny “CoAs” plots, where levels raised up to 55 kg N ha^−1^, 6 kg P ha^−1^, and 33 kg K ha^−1^ (Table 2, Table S1). “Comp” and “CoAs” treatments showed higher plant uptake on the sunny plots, while for “Ash” plant uptake was doubled on the shaded plots (Table 2). For “Ctr” plots, the uptake was also slightly higher in the shaded plots.

**Table 2:** Mean values with standard deviations for plant uptake in shaded and sunny plots

Treatment	Shaded plots			Sunny plots		
	N uptake (kg/ha)	P uptake (kg/ha)	K uptake (kg/ha)	N uptake (kg/ha)	P uptake (kg/ha)	K uptake (kg/ha)
Compost	5.77 ± 0.74	0.63 ± 0.05	4.15 ± 0.19	18.13 ± 3.00	2.01 ± 0.17	11.69 ± 0.43
CoAs	25.93 ± 2.69	3.07 ± 0.65	16.20 ± 1.18	55.62 ± 4.94	6.40 ± 0.42	33.08 ± 3.55
Ash	17.49 ± 1.00	2.05 ± 0.24	12.34 ± 0.97	9.40 ± 1.05	1.02 ± 0.13	6.24 ± 0.45
Control	8.30 ± 0.77	0.58 ± 0.15	6.57 ± 1.18	6.12 ± 2.11	0.47 ± 0.25	4.93 ± 0.72

### 3.2 Soil physicochemical fertility and microbial activity

**Figure 2:**
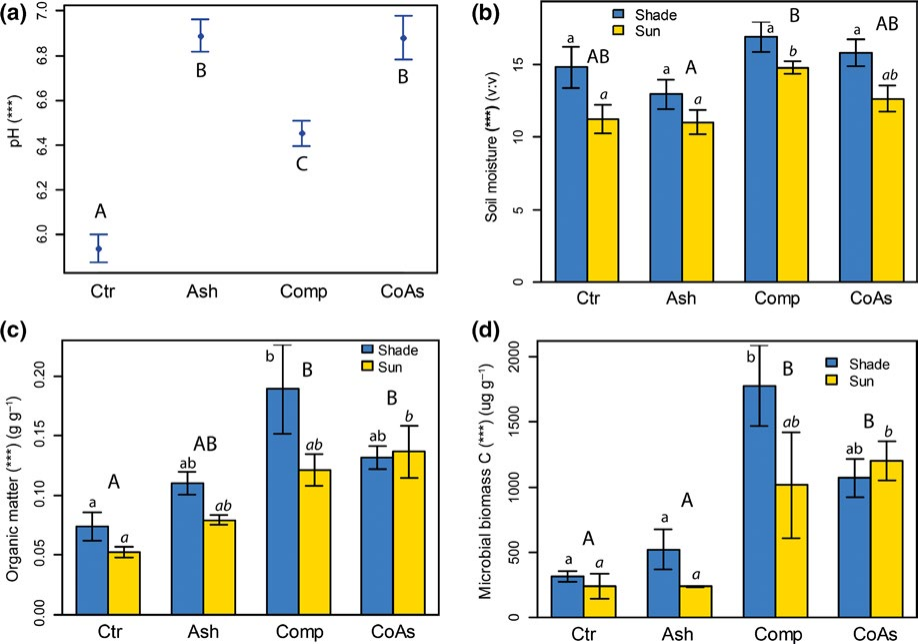
Mean (±standard errors) soil pH (a), moisture (b), organic matter (c), and microbial biomass (d) according to the different treatments. For abbreviations, asterisks and letters, cf. Figure 1. Results for shaded and sunny plots are confounded for (a) because the effect was not significant

After corn harvest, the different soil amendments did not display significant differences in soil nutrient concentrations, except for K concentration which was higher in “Comp” and “CoAs” plots (Fig. S1 and Table S1). The amendment of soil with ash and compost changed its acidity from moderately acid in the “Ctr” plots to slightly acid in “Comp” plots to neutral in “Ash” and “CoAs” plots (Figure 2). No difference in soil pH was found between the shaded and sunny plots (Table S1). Soil moisture, microbial biomass, and organic matter were significantly higher in shaded plots than in sunny plots (*p *≤* *.05, Table S1). “Comp” and “CoAs” treatments resulted generally in a significantly higher soil moisture, carbon microbial biomass, and organic matter, compared to “Ash” and “Ctr” treatments. “Comp” treatment increased soil moisture by 15%–35% and organic matter by 50%, as well as multiplied microbial biomass by 4–5 times compared to “Ash” and “Ctr” plots. “CoAs” treatment exhibited slightly lower soil variables than “Comp” treatment; however, the difference between the two treatments was not statistically significant.

**Figure 3:**
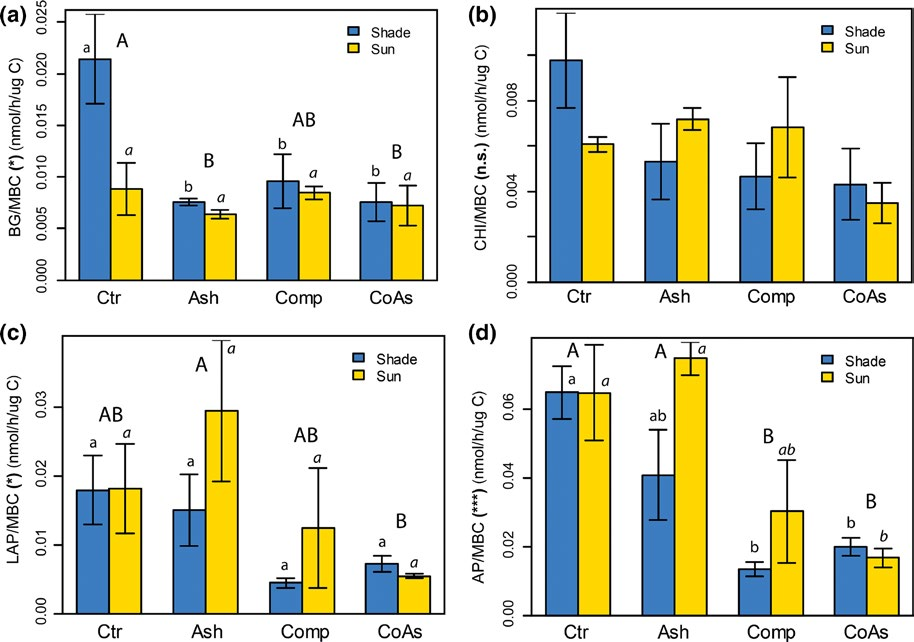
Mean (±standard errors) enzymatic activities normalized by carbon microbial biomass (MBC) according to the different soil treatments, (a) beta‐glucosidase (BG), (b) chitinase (CHI), (c) leucine aminopeptidase (LAP), (d) acid phosphatase (AP). For abbreviations, asterisks and letters, cf. Figure 1

The enzymatic activities indicate a generally higher microbial activity in “Ash” and “Ctr” plots. Enzymatic activities significantly differed according to the soil amendment treatments (Figure 3 and Table S1), except for CHI (*p *>* *.05). AP (*p *≤* *.001) and LAP (*p *=* *.015) activities were generally higher in the “Ctr” and “Ash” plots while BG showed only a significantly higher value for the “Ctr” plots compared to other soil amendments (*p *=* *.012). Moreover, AP and LAP were correlated to P uptake (*r* = .44, *p *=* *.03) and N uptake (*r* = .52, *p *=* *.012), respectively.

### 3.3 Underlying relationships between crop yield and soil fertility drivers

**Figure 4:**
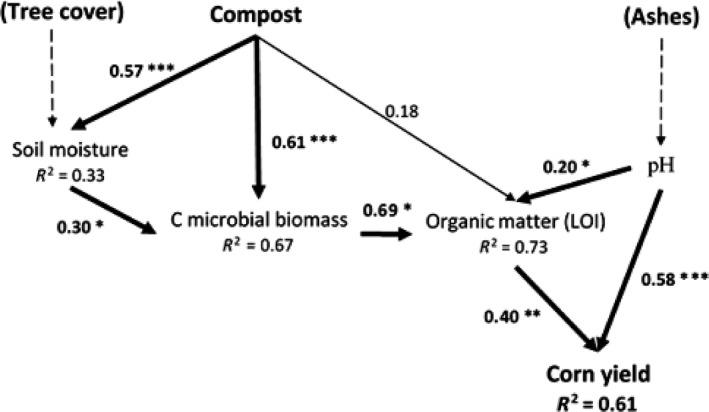
Structural equation model for the effect of compost and ash on corn yield. Bold arrows show significant relationships (pathways) between variables (for asterisks, cf. Figure 1), the thin arrow indicates a nonsignificant relationship, and numbers next to arrows show standardized parameter estimates (i.e., standardized regression weights). Dotted arrows indicate existing relationships which have not been integrated in the model for stability reason. Squared multiple correlations (R2) for the predicted/dependent factor are given below the dependent variables. All model fit indices were good: chi‐square = 7.65, *p *=* *.36, GFI = 0.91, RMSEA = 0.06, SRMR = 0.07 and AIC = 35.6

Independently of tree cover, structural equation modeling showed that two distinct pathways explained corn yield (*r* ^2^ = 0.61; Figure 4). The first pathway was driven by compost amendment that directly increased both soil moisture (path = 0.57) and carbon microbial biomass (path = 0.61). Increasing soil moisture also promoted carbon microbial biomass (path = 0.30) but in a lesser extent that the direct effect of compost (Figure 4). Carbon microbial biomass positively and strongly increased soil organic matter (path = 0.69), which in turn promoted corn yield (path = 0.40). The second pathway highlighted the direct effect of increasing pH due to ash amendment on corn yield (path = 0.58). In sum, our model showed that high organic matter content and a rise of pH from moderately acid to neutral were the main drivers of corn yield.

## 4 DISCUSSION

Our findings show that a combined soil amendment of ashes and compost multiplies corn yield by four to five compared to compost or ash alone, respectively. Our analysis based on structural equation modeling allowed interpreting the complex interactions between compost and ashes on soil fertility processes driving corn yield. Although ultimate causality could not be established with this method, it, however, highlights that each amendment enhances distinct, but complementary, properties of soil fertility, resulting in a multiplicative effect on corn yield.

### 4.1 The ashes’ pathway

Ash amendment is known for its alkalinity properties which rise the soil pH (Demeyer et al., 2001). In our study, ash pH was moderately basic, being of 8.6, while in other conditions, it could attain values up to 13.5 (Etiégni & Campbell, 1991). Wood, at the center of the pile, burns at higher temperatures and longer than wood on the edges, which influences the alkalinity of the ashes; higher temperatures produce more alkaline materials (Demeyer et al., 2001). Then, the modification of soil pH following ash amendment changes soil nutrient availability, in particular phosphorus. In tropical acid soils, P availability is mostly controlled by the strong adsorption capacity of iron and aluminum minerals, which tend to occlude phosphorus (Frossard, Brossard, Hedley, & Metherell, 1995). An increase in pH favors the release of those occluded phosphorus forms into the soil solution and increase phosphorus availability (DeBano & Klopatek, 1988).

We found that the ratio enzyme activity to microbial biomass increased in ash‐alone amendment and controls, especially for LAP and AP. Enzyme activities are indicators of differences in soil conditions and response to modification of nutrient supply (Wallenstein, McMahon, & Shimel, 2009; Weedon et al., 2012). LAP is linked with N soil supplies, while AP is linked with P soil supplies (Sinsabaugh et al., 2008). Microorganisms require an appropriate nutrient supply to uphold essential physiological processes for their survival, like the synthesis of proteins or nucleic acids. When one or several nutrients become limiting, microorganisms focus their activity toward the acquirement of these nutrients for their survival, rather than their multiplication (Liao & Xie, 2007). Hence, in our system, enzymatic activities suggest that in “Ctr” and “Ash” treatments, the soil is particularly depleted into available N and P for microbes and plants as compared to the treatments “Comp” and “CoAs.”

### 4.2 The compost pathway

Compost enhanced soil moisture, organic matter and microbial biomass, in “Comp” and “CoAs” treatments compared to the “Ctr” and “Ash” treatments. By selecting three local tree species with a very high nutrient content (Raharimalala, 2011), we produced an efficient compost. This suggests that the effects of compost on soil properties could be reduced with the use of other composting material, as reported by Lima, de Queiroz, and Freitas (2004) who showed that compost made with thoroughly selected vegetal waste increased by 80% the biomass of corn plants compared to a nonselected compost.

Using compost in agriculture usually helps in preserving long‐term soil fertility with the improvement of soil microbial biomass and activity, as well as, providing slow‐released nutrients in accordance with crop requirements (Mbau et al., 2014; Zhang et al., 2016). However, despite the observed enhancements of soil fertility through compost, crop yields remained similar between “Ash” and “Comp” treatments. Only “CoAs” treatment led to significantly greater corn yields with 1.5 t/ha instead of instead of 0.25 t/ha for ashes and 0.35 t/ha for compost. These findings show that, on acid soils, compost amended with ashes improves significantly both soil fertility and plant growth. This synergic effect is probably due to the pH elevation effect of ashes on both organic matter content amended by compost and corn yield, as supported by the SEM. Indeed, Kuba, Tschöll, Partl, Meyer, and Insam (2008) and Bougnom et al. (2010) found similar results in other systems and demonstrated that compost amended with industrial ashes on acid and nutrient‐deficient soils resulted in better plant cover and soil microbiological properties, due to the positive effect of ashes on pH (Gabhane et al., 2012). Consequently, our results suggest that a continuous application of compost in combination with ashes could be a suitable method for the reclamation of most abandoned fields in Madagascar.

### 4.3 Trees double‐game

Trees play simultaneously a role of soil protector and light competitor. The first role benefits corn plants, while the second prevents their growth. This double effect is revealed in our study, by the contrasting results found between “Ash” treatment and the other treatments. Indeed, yield was decreased on shaded plots (50% tree cover) compared to sunny plot (0% tree cover) for all treatments, except “Ash.” Ashes, because of their black color, diminish soil albedo and increase soil temperature (Usowicz, Lipiec, Łukowski, Marczewski, & Usowicz, 2016). Thus, on sunny plots, soil moisture was reduced while on shaded plots, soil moisture has probably been retained longer at the end of the rainy season, increasing potentially the corn growing season for a while (Çakir, 2004; Payero, Melvin, Irmak, & Tarkalson, 2006). Indeed, plants suffering from water stress cease their growth because nutrient uptake is not possible anymore (Sardans & Peñuelas, 2012). At the opposite, for the other treatments, the shade of the trees impacted negatively corn growth by probably preventing an optimum photosynthesis. When intercropping trees and corn, Reynolds, Simpson, Thevathasan, and Gordon (2007) assessed a significant reduction in solar radiation, associated with a reduced yield, for corn plants growing at 2 m of the trees. Tree cover should be therefore kept to a minimum and corn plants should be planted at a higher distance. Carrière et al. (2002) showed in South Cameroon that 3–7 trees for a field of 0.8 ha are enough to improve the slash‐and‐burn agricultural system.

### 4.4 Further considerations on up‐scaling

A violent storm occurred during the time of our experiment: from 17 to 19 January 2015, heavy rains flooded the Kirindy forest due to the cyclone Chedza. Such an extreme event may have affected the results of the experiment and considerably reduced the expected grain yield of the different treatments. Gay‐des‐Combes et al. (2017) showed that such storm can decrease the grain yield from 4 to 1 t/ha and lower the nitrogen and phosphorus soil concentrations by at least 50%. Given what precedes and considering that the study was performed over only 1 year and on one particular type of soil, the proposed reclamation strategy needs to be checked over longer periods, and multisites before the results could be generalized. However, because of the cyclone, our experiment faced one of the worst‐case scenarios in terms of nutrient depletion. Thus, most probably, similar or better outcomes would be reached if the proposed cultural method is re‐tested in better conditions in terms of soil fertility or climate.

On the long term, the increase in labor requirement, due to the compost fabrication and maintenance, may halt that technique to be endorsed by the farmers. However, compost is very demanding in labor only on a short period of time, namely for green material collection and preparation. If that task can be performed at the beginning of the rainy season, when maize has been sown, it may suit with farmer's cultural calendar. One might also argue that improved production in shifting cultivation will not reduce deforestation, but rather accentuates deforestation to increase production over a larger scale. We observed that for 1 day of work invested in compost production, only c. 700 m^2^ of crop field can be amended with compost (Gay‐des‐Combes, unpublished data). Considering that most farmers possess 1–2 ha (Dirac Ramohavelo, 2009), they would need about 14–35 days to produce enough compost to cover their entire fields. In consequence, to limit labor constraints and to avoid further damage on the primary forest, we would rather recommend compost as a tool to sustain cultivation on the most degraded fields, rather than a solution for large scale fields.

As a follow‐up of this research, composting workshops have been performed with farmers in Beroboka and two other neighboring villages (Marofandilia and Kirindy) between May 2015 and December 2016. As slash‐and‐burn agriculture is embedded in traditions and spirituality, the developed methodology, which stays close to traditional practices, was expected to meet a high social acceptance (Desbureaux & Brimont, 2015; Jarosz, 1993). Indeed, five hundred farmers participated to the workshops, corresponding to about 13% of the population of the villages. This local interest called for further social and ecological research on the refinement and validation of that alternative slash‐and‐burn practice.

## 5 CONCLUSION

A combination of wood ashes and compost seems to be a good solution for recovering tropical degraded soils but requires further investigation before generalization. In our experiment, it increased corn yield by 4–5 compared to traditional slash‐and‐burn agriculture. Thus, the proposed solution is original because it considers the deeply socially rooted slash‐and‐burn practice as an acceptable solution if it is well matched with soil organic matter enrichment, whereas it is usually dismissed as destructive. Recognizing this type of agriculture as a cultural heritage and trying to modify it to make it sustainable paves the way toward a more integrative vision of traditional agriculture along with tropical forest management.

## AUTHOR CONTRIBUTIONS

JMG and AB conceived the experimental design; JMG, CSC, LF collected field data; CSC, RTEM, and MSA either performed or gave advices on laboratory analyses and undertook preliminary statistical analyses; JMG and VEJJ conceived the structural equation model; JMG and BMJR wrote the first draft of the manuscript and finalized the statistical analyses; EF and AB considerably improved the manuscript. All authors contributed to all drafts and gave final approval for publication.

## CONFLICT OF INTEREST

None declared.
