# Supplementary material for: Tropical soils degraded by slash‐and‐burn cultivation can be recultivated when amended with ashes and compost
Source: Ecol Evol. 2017 Jun 12;7(14):5378–88. doi: 10.1002/ece3.3104 (PMC5528233; doi:10.1002/ece3.3104)

**Supporting information**

**Detailed methodology for the laboratory analyses**

*Plant nutrient content and uptake*

Nitrogen (N), phosphorus (P) and potassium (K) content of the corn leaves and grains were assessed by digesting the plant samples as described by Wolf (1982). In short, c. 0.05 g of air-dried and ground (0.1 mm) plant biomass was digested in 3 mL of H_2_SO_4,_using selenium (Na_2_SO_4_Se) as catalyst, and heated up to 420^o^C for 15 min. The volume of extract was diluted with distilled water and then used for determination of NPK in plant biomass. Final nutrient concentrations were corrected for the dry weight of the plant biomass obtained after drying a sub-sample at 105 °C for 24 hours. Further, potassium (K) was determined by atomic absorption spectrophotometry (Solaar 969, ThermoOptek). Total nitrogen (N) and phosphorus (P) concentrations were determined by, respectively, the blue-indophenol method and the molybdovanadate method using a continuous flow autoanalyser (FlowSys, Systea, Anagni, Italy). Standard reference material (NIST Citrus leaves 1572, National Bureau of Standards) was analysed along with the samples in order to ensure accuracy within 5% of known K, N and P concentrations. Plant uptake in aerial parts was calculated as the sum of the product of leaf and grain nutrient content and the respective leaf and grain dry weight.

*Soil, ash and compost parameters*

Total nitrogen (N) and carbon (C) were measured on milled soil with a standard CHN analyser (Dumas method). **The pH was assessed in a 1:2.5 soil:water suspension (v:v)** (Allen 1989). Cation exchange capacity (CEC), exchangeable K and inorganic nitrogen (NO_3_ and NH_4_) were measured by extraction: CEC and K with Cobalt-hexamine solution (Co(NH_3_)Cl_3_ 0.0166 M) and inorganic nitrogen with KCl (1 M). The extractions were done on 10 g of dry soil with 40 ml of solution. Then, they were shaken for one hour at 120 rpm and filtered at 0.45 µm. Cation exchange capacity and exchangeable potassium were read on **a plasma atomic emission spectrometer (Shimadzu ICPE-9000).** Inorganic nitrogen forms **were** analysed using a continuous flow analyser. Plant phosphorus availability was measured with an anion-exchange resin (Hedley, Steward & Chauhan 1982). Five grams of dry ground soil samples were shaken during the night (16h) with resin membrane strips (bicarbonate charged) in a miliQ solution. Further, resins were eluted with a NaCl-HCl 0.5M solution to extract phosphorus (Kouno, Tuchiya & Ando 1995). Phosphorus concentrations were measured with the malachite green method and by inserting the samples in a Shimadzu 1800 UV-Vis spectrophotometer (Ohno & Zibilske 1991). Lastly, organic matter was measured through loss of ignition at 600°C. **All measures were performed on the initial soil of the experimental sites before the experiment started, on the ashes and compost (at maturation) used in the experiment, and on soil samples of every plot at the end of the experiment.**

*Soil respiration, microbial biomass and soil enzyme activity*

Microbial C biomass was measured with a **chloroform fumigation followed by 0.5 M K_2_SO_4_ extraction** (Vance, Brookes & Jenkinson 1987). Shortly, soil samples were divided in two 5g sub-samples. One of each set of sub-samples was chloroform fumigated in a desiccator over 24 hours in the dark, while the other sub-samples remained in similar conditions but without chloroform. Then, an extraction was done on both sets of sub-samples. After addition of 25ml 0.5M K_2_SO_4_, all samples were shaken at 120 rpm during 1 hour. Samples were finally filtered at 150mm, and C concentration was measured on a Shimadzu TOC-V analyzer. Microbial biomass was calculated by the difference between chloroform fumigated and control samples and corrected with Kc factor (Kc=0.45, Beck et al. 1997).

As a proxy for soil activity, we measured the activity of four enzymes involved in C, N and P cycling (Sinsabaugh et al., 2008). We used substrates labelled with the fluorophores 7-amino-4-methylcoumarin (MUC) or methylumbelliferone (MUB) to quantify the relative activity (i.e. enzyme activity under optimal and saturating substrate conditions) of enzymes responsible for the hydrolysis of one peptide (Leucine amino-peptidase, LAP), two carbohydrates (β-glucosidase, BG; Chitinase, CHI), and one phosphatase (acid phosphatase, AP; all substrates supplied Sigma-Aldrich Switzerland). Extracellular enzymes were extracted following (Criquet *et al.* 2000) and analysed in microplates. For each sample, 4 pseudo-replicate wells were included in a 96 well microtiter plate. Four pseudo-replicate wells containing boiled extracts (3h at 90°C) were also performed as a control to take into account the quenching effect (Burns *et al.* 2013). Each wells received 38 µL of enzyme extract and 250 µL of substrate. Microplates were then incubated at 25°C for 3h. Fluorescence was monitored spectrophotometrically with an excitation wavelength of 365 nm and emission detection at 450 nm (Biotek, SynergyMX). All enzymatic activities were converted to nanomoles per gram dry weight per min (nmol.min^-1^.g^-1^ DM).

**Supplementary references**

Beck, T., Joergensen, R.G., Kandeler, E., Makeshin, E., Nuss, E., Oberholzer, H.R. & Scheu, S., 1997. An inter-laboratory comparison of ten different ways of measuring soil microbial biomass C. *Soil Biology & Biochemistry*, 29(7), pp.1023–1032.

Burns, R.G., DeForest, J.L., Marxsen, J., Sinsabaugh, R.L., Stromberger, M.E., Wallenstein, M.D., Weintraub, M.N., Zoppini, A., 2013. Soil enzymes in a changing environment: Current knowledge and future directions. Soil Biol. Biochem. 58, 216–234. doi:http://dx.doi.org/10.1016/j.soilbio.2012.11.009

Criquet, S., Farnet, A.M., Tagger, S. & Petit, J.L. (2000) Annual variations of phenoloxidase activities in an evergreen oak litter: influence of certain biotic and abiotic factors. *Soil Biology and Biochemistry*, **32**, 1505–1513.

Kouno, K., Tuchiya, Y., Ando, T., 1995. Measurement of soil microbial biomass phosphorus by an anion exchange membrane method. Soil Biol. Biochem. 27, 1353–1357. doi:http://dx.doi.org/10.1016/0038-0717(95)00057-L

**Table S1** Summary of split-plot models. Significant values are in bold.

|  | Tree cover, shade (S) | | Soil amendment (T) | | SxT | |
| --- | --- | --- | --- | --- | --- | --- |
| Parameter | F | *P* | F | *P* | F | *P* |
| Height | 0.727 | 0.484 | 28.055 | **<0.001** | 3.918 | **0.037** |
| Grain yield | 1.782 | 0.314 | 17.998 | **<0.001** | 3.471 | **0.051** |
| Plant K uptake | 0.478 | 0.498 | 5.070 | **0.037** | 0.013 | 0.910 |
| Plant N uptake | 0.405 | 0.533 | 5.715 | **0.028** | 0.050 | 0.825 |
| Plant P uptake | 0.223 | 0.642 | 8.046 | **0.011** | 0.027 | 0.872 |
| pH | 0.22 | 0.68 | 20.82 | **<0.001** | 0.72 | 0.56 |
| Soil moisture | 27.617 | **<0.001** | 10.478 | **<0.001** | 0.614 | 0.617 |
| Organic matter | 106.7 | **0.009** | 11.170 | **<0.001** | 0.933 | 0.455 |
| Soil inorg. N | 4.855 | 0.158 | 1.381 | 0.296 | 1.188 | 0.355 |
| Soil resin P | 4.856 | 0.158 | 1.381 | 0.296 | 1.188 | 0.355 |
| Soil ex K | 1.74 | 0.32 | 4.31 | **0.028** | 2.22 | 0.139 |
| Microbial biomass C (MBC) | 58.92 | **0.017** | 18.773 | **<0.001** | 2.246 | 0.135 |
| Activity AP/MBC | 4.998 | **0.045** | 16.503 | **<0.001** | 2.306 | 0.128 |
| Activity LAP/MBC | 3.365 | 0.092 | 5.275 | **0.015** | 1.478 | 0.270 |
| Activity BG/MBC | 3.598 | 0.082 | 5.613 | **0.012** | 2.512 | 0.108 |
| Activity CHI/MBC | 0.002 | 0.965 | 2.040 | 0.162 | 1.136 | 0.374 |

**Table S2** Components of hypothesis represented by structural equation model of Figure 3

| **Path** | **Hypothesized mechanisms based on *a-priori* knowledge (cf. table S1)** |
| --- | --- |
| Compost -> Soil moisture | Compost increases soil moisture. |
| Compost -> Microbial biomass | Compost increases microbial biomass. |
| Tree cover -> Soil moisture | Tree cover increases soil moisture. |
| Soil moisture -> Microbial biomass | Soil moisture increases microbial biomass. |
| Compost -> Organic matter | Compost provides organic matter to the soil. |
| Microbial biomass ->Organic matter | Microbial biomass degrades litter and increases organic matter. |
| Organic matter -> Corn yield | Organic matter increases plant available nutrients and thus enhances corn yield. |
| Ash -> pH | Ash rises soil pH. |
| pH -> Organic matter | pH enhances organic matter. |
| pH -> Corn yield | pH favors plant growth when optimal. |

**Figure S1** Soil inorganic N, resin P and exchangeable K concentrations after harvest of corn.
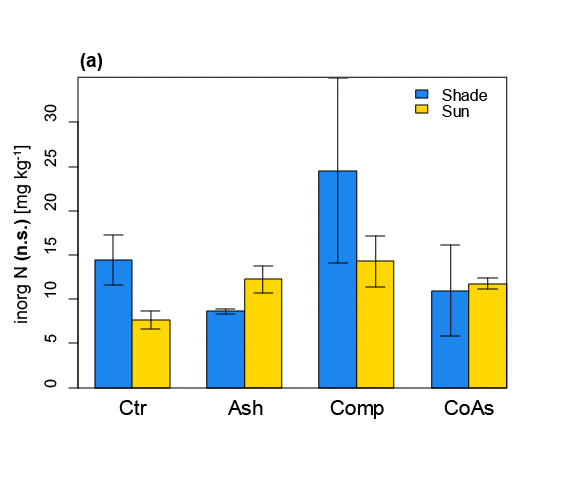

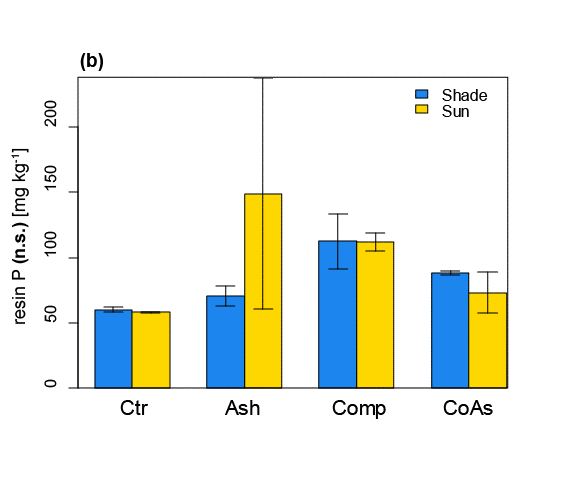

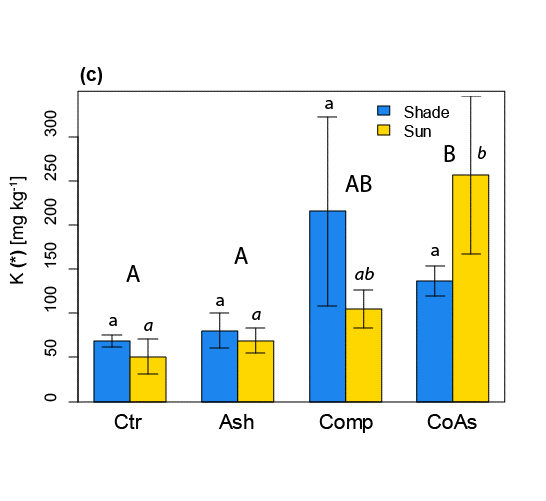

Supplement: Supplementary file 1 [file ECE3-7-5378-s001.docx]
